# 1D kinetic modelling of the JET SOL with tungsten divertor plates

**DOI:** 10.1016/j.jnucmat.2013.01.108

**Published:** 2013-07

**Authors:** D. Tskhakaya, M. Groth, JET EFDA contributors

**Affiliations:** a JET-EFDA, Culham Science Centre, OX14 3DB Abingdon, UK; b Association EURATOM-ÖAW, University of Innsbruck, Technikerstrasse 25/II, A-6020 Innsbruck, Austria; c Aalto University, Association EURATOM-Tekes, Otakaari 4, 02015 Espoo, Finland

## Abstract

In this work a fully kinetic model of the JET SOL with tungsten divertor plates has been developed. It includes the dynamics of main-ions (D^+^) and electrons, the neutrals (D, C, W) and the impurity particles (C^+m^, W^+n^). Our simulations show extremely low concentration of W impurity. We identify two reasons which are responsible for this effect: (1) for low temperature divertor plasma the energy of most of the main-ions and the impurities in a low-ionization state impinging the divertor plates is below the W-sputtering threshold energy; (2) with increasing temperature the W-sputtering increases, but the potential drop across the divertor plasma increases too, so that most of the W ions are reabsorbed at the divertors.

## 1 Introduction

Tungsten is becoming a common divertor material for our day and future tokamaks. As a result, the development of the corresponding SOL models has become one of most important topics in fusion plasma research. In the present work we model tungsten generation and transport along the field lines in the JET SOL using fully kinetic approach: the plasma (e, D^+^), the neutral (D, C, W) and the impurity (W^n+^, C^m+^) particles are treated kinetically.

The simulations of this type are extremely CPU-intensive. There are several reasons for using such modeling. It has been demonstrated that kinetic effects can dominate in the high recycling plasma even if there are only common impurities like carbon (see [1], [2], [3], [4], [5] and references there). Introduction of massive high-z impurities complicates the problem, so that the kinetic effects can become essential. Here we mention two additional effects.

First of all, massive high-z impurities (like tungsten) cannot be treated as trace impurities. E.g. the friction force between different ionized states of W, $R_{{\text{W}}^{+\text{k}}{\text{W}}^{+\text{n}}}$, can be of the same order as the friction force between W and main D ions, $R_{{\text{W}}^{+\text{k}}{\text{D}}^{+}}$, [6]:

(1) $$\underset{n\neq k}{\sum }R_{{\text{W}}^{+\text{k}}W^{+\text{n}}}/R_{{\text{W}}^{+\text{k}}{\text{D}}^{+}}∼\sqrt{M_{\text{W}}/M_{\text{D}}}\approx 10\underset{n\neq k}{\sum }n^{2}c_{{\text{W}}^{+\text{n}}}\text{,}$$

where *M*_W,D_ are particle masses and $c_{{\text{W}}^{+\text{n}}}$ is the W^+n^ concentration. As we see, if $\sum_{n\neq k}n^{2}c_{{\text{W}}^{+\text{n}}}∼0.1$, then the friction force between W ions cannot be neglected. We note that due to lower mass ratio this effect is practically negligible for light impurities.

**Fig. 1:**
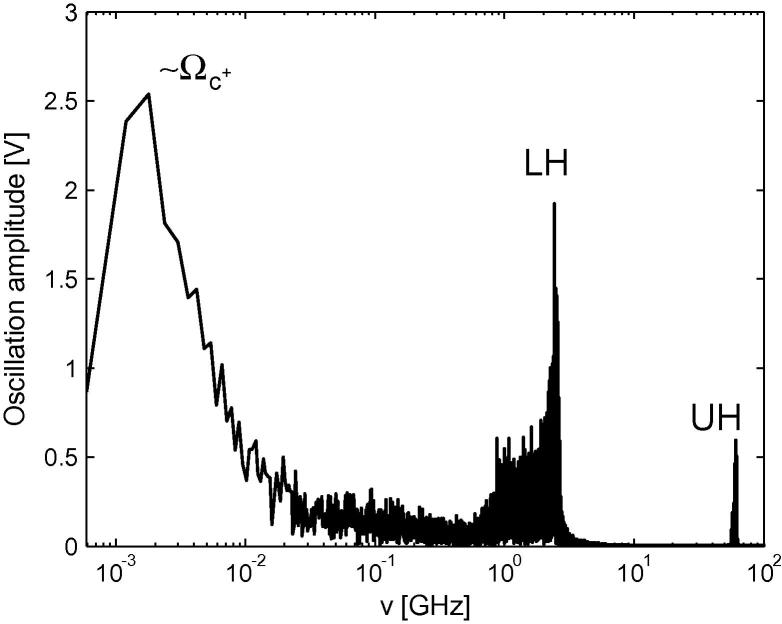
Potential oscillation spectrum at the magnetic presheath entrance in the outer divertor plasma.

The second effect is related to the tungsten sputtering, which is strongly coupled with the divertor plasma parameters and extremely sensitive to the energy of ions impinging at the divertor plates. It is usually assumed that these ions are accelerated in a constant (in time) sheath potential drop ∼3*T*_e_/*e* (*T*_e_ is the electron temperature). In reality the potential oscillates around this average value, which may accelerate resonant ions up to energies more than 3*T*_e_. In Fig. 1 is plotted the oscillation spectrum of the potential at the magnetic presheath entrance in the outer divertor plasma (the divertor potential is set to zero). The maximum at low frequency is near to C^+^ cyclotron and the other two correspond to the lower and upper hybrid wave frequences [7]:

(2) $$\omega_{\text{LH,UH}}={\left[\frac{\Omega_{\text{e}}^{2}+\omega_{p}^{2}}{2}\pm \sqrt{\frac{{\left(\Omega_{\text{e}}^{2}+\omega_{\text{p}}^{2}\right)}^{2}}{4}-\omega_{\text{p}}^{2}\Omega_{\text{e}}^{2}\sin^{2}\theta }\right]}^{1/2}\text{,}$$

where *Ω*_e_ and *ω*_p_ the electron cyclotron and plasma frequencies; *θ* is the angle between the magnetic field and the divertor surface. Although the amplitude of these oscillations is lower than the average potential (∼110 eV), it is not obvious that the additional energy gain by resonant ions is negligible. Moreover, the tungsten atoms can be ionized near to the divertor plates and the probability to return back to the plates strongly depends on the electric field (and its oscillations) in the sheath. As we will see below, exactly this redeposition is responsible for significant reduction of the effective W-sputtering yield.

These examples indicate that for realistic self-consistent modeling of the W generation and transport in the SOL a fully kinetic approach is required.

## 2 Description of the SOL model

**Fig. 2:**
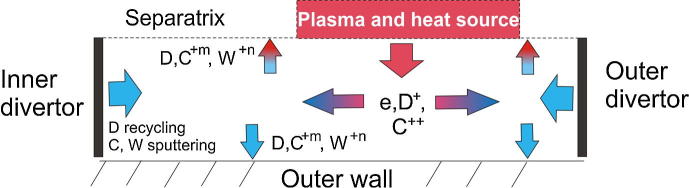
PIC simulation geometry.

For the simulation we have updated the original Particle in Cell (PIC) Monte Carlo (MC) code “BIT1” by including new physics and optimization of number of numerical routines. BIT1 is a quasi-2D massively parallel kinetic code for simulation of the SOL [8]. The simulation geometry represents a rectangular box two sides which correspond to the divertor plates and the other two to the separatrix and to the outer wall (see Fig. 2). The plasma, the neutral and the impurity particles are treated in 1D3V, 2D3V and quasi-2D3V approximation, respectively (*n*D*m*V means *n*-dimensional in usual and *m*-dimensional in velocity space). Hot (120–250 eV) plasma (e, D^+^), impurity (C^++^) and heat source correspond to the particle and heat transport across the separatrix. After the injection plasma and impurity particles propagate along the magnetic field towards the divertor. The particle absorbed at the divertor plates cause injection of secondary particles (secondary electrons, D, C and W atoms). These atoms interact with the plasma in a nonlinear way. Atoms reaching the radial boundaries of the system (i.e. the separatrix and the outer wall) are removed from the system. Impurity ions, C^+m^, W^+n^, are also removed from the simulation with the probability corresponding to the anomalous cross-field diffusion coefficient $D_{⊥}∼1\;{\text{m}}^{2}/\text{s}$ and cross-field gradient length ∼1 cm. To keep quasineutrality the corresponding number of electrons is removed together with the impurity ions. The strength of the particle and heat sources and the temperature of the incoming particles are adjusted to match the experimentally observed plasma density and electron temperature in the upstream SOL. For other details of the simulation see [1], [5].

All the collision operators used in the code are nonlinear and conserve the particle number, the momentum and the energy. The number of simulated particle species is limited (practically) by available atomic and PSI (Plasma-Surface Interaction) data. In the given simulations we included C^+m^, W^+n^, *m* = 0,…, 2, *n* = 0,…, 11, impurity ions, hence together with electrons and the main ions there 15 different types of charged particles interacting with each other. In the simulations we do not observe highly ionized tungsten ions (*n* > 4), so that the number collision types is reduced significantly.

The threshold energy for W sputtering due to D impact is too high to produce any reasonable amount of W [9], [10] (our test simulations also confirm this). In realistic plasmas, light impurities, like C and Be having relatively low W-sputtering threshold energies, are the catalysts for W production. Both C and Be have comparable mass and threshold energies, and may be equally used in the simulations for production of W. In order to simplify the model we consider only C impurities. The original BIT1 included all necessary atomic and PSI processes for simulation of e, D^+^, C^+m^ plasmas. Hence, the only missing part was the tungsten-related atomic and PSI processes.

Implementation of atomic processes. For the atomic processes in the BIT1 code we consider single and double electron-impact ionizations of W^+n^, for *n *< 11 and *n *< 7, respectively. The corresponding cross-sections are taken from [11], [12]. Some of cross-sections are given for energies below 1 keV. In this case we extrapolated the cross-sections according to the expression [13]:

(3) $$\sigma =\frac{A\ln(E)+B}{E}\text{.}$$

**Fig. 3:**
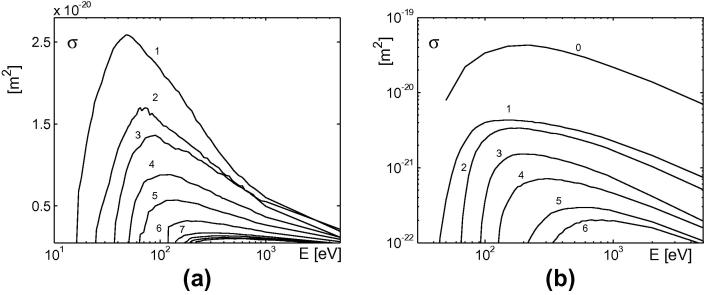
Single (a) and double (b) electron-impact ionization cross-sections for tungsten implemented in the BIT1 code. Numbers indicate initial ionization state.

The obtained cross-sections are plotted in Fig. 3. The after-collision electrons are assumed to be izotropically scattered.

**Fig. 4:**
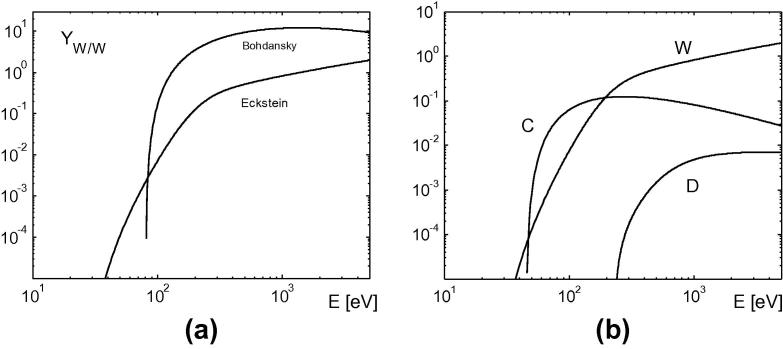
W-sputtering yield for normal impact of different particles. (a) W self-sputtering yield from different sources; (b) yields implemented in the BIT1 code.

Implementation of PSI processes. Contrary to the ionization-cross sections there is a large spread in tungsten-related PSI data. E.g. the tungsten self-sputtering yield given in [9] is too large and results, according to our test simulations, in unphysically high W concentrations. During the simulation W density reached $0.5\times 10^{20}\;{\text{m}}^{3}$ and was rapidly increasing, so we stopped the run. Eckstein in [10] proposes more realistic sputtering yields (see Fig. 4), which are implemented in the BIT1. Unfortunately, in [10] the W sputtering yields due to carbon impact are missing, hence we used the data from [9] with the corrected threshold energy (45 eV) considered in [14].

The probability that after ionization the sputtered W returns back to the divertor strongly depends on the distribution of sputtered W atoms. Hence, we implemented the following sputtered-W-distribution model. For D and W impact we use the fit function from [15]:

(4) $$f_{\text{w}}(E)=\frac{2a^{2}E_{\text{s}}E}{(E+E_{\text{s}}{)}^{3}}\text{,}\;a\equiv 1+\frac{(M_{1}+M_{2}{)}^{2}}{4M_{1}M_{2}}\frac{E_{\text{s}}}{E_{0}}\text{.}$$

where *M*_1_ and *M*_2_ are the atomic masses of the target and projectile atoms (ions); *E*_0_ and *E*_s_ are the surface binding and impinging+ particle energies. For the C induced W sputtering we use a simple model:

(5) $$f_{w}(E)=\left\{\begin{array}{c} \text{const,}\;\text{for}\;E⩽E_{\max}=10\;\text{eV,}\\ 0\text{,}\;\text{for}\;E>E_{\max}\text{.} \end{array}\right)$$

The angular distribution for the both models is the “cosine” one: $\cos(\alpha )=\sqrt{\mathrm{Random}\;\mathrm{Number}}$, where *α* is the angle between the velocity of injected W and the normal to the divertor plate.

During the simulation we use 60,000 cells along the poloidal direction. This allows finest resolution in space down to the Debey length and electron gyro-radius. Each run took in average 20,000 CPU hours on 1024 processors, all together (including test runs) about 300,000 CPU hours have been consumed.

## 3 Simulation results

During the simulations we adjust the plasma and the heat source parameters to match the experimentally observed upstream SOL density, *n*_u_, and electron temperature, *T*_e,u_. For reference we consider the shots #81472, #81478 and #81484 with $n_{u}∼1.5\text{"–"}1.8\times 10^{19}\;{\text{m}}^{-3}$, $T_{e\text{,}u}∼45\text{"–"}75\;\text{eV}$. Simulation parameters were chosen in a way to match these upstream data. We made three sets of simulations:

1. High temperature case ($T_{e\text{,}u}∼65\;\text{eV}$) with relatively strong heat source.
2. Low temperature case ($T_{e\text{,}u}∼45\;\text{eV}$) with 2.5 times weaker heat source.
3. The case as 1. with the additional injection of ∼100 eV C^++^ ions from the particle source. In this way we simulate influx of hot carbon ions from the pedestal.

Low temperature carbon particles originating from different plasma-facing-components are modeled via injection of C atoms from the divertors with the fixed flux 10^21^ m^2^/s. C atoms are assumed to be in thermal equilibrium with Franck–Condon distributed D atoms and have the temperature 2 eV.

**Fig. 5:**
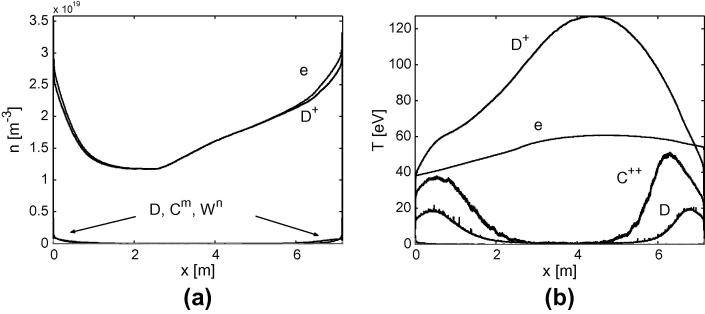
Poloidal profiles of the density (a) and the temperature (b) in the SOL for the case 1.

**Fig. 6:**
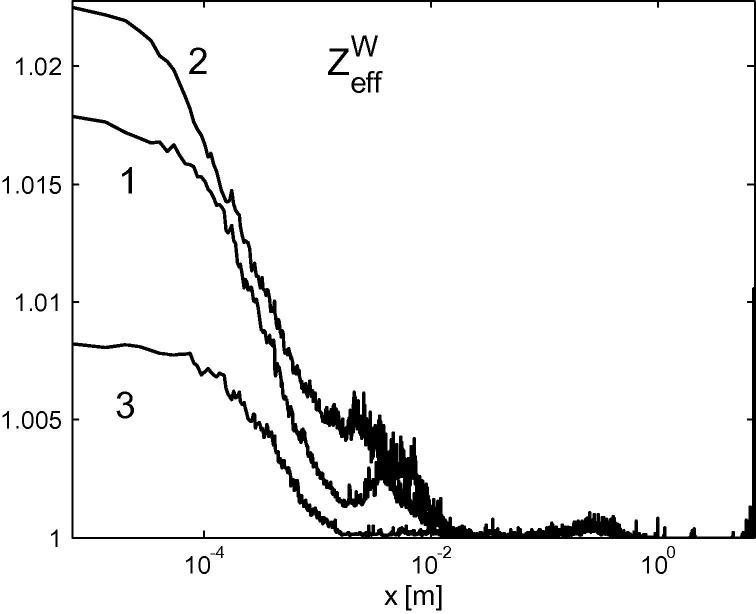
Poloidal profiles of $Z_{\text{eff}}^{W}$. The numbers “1”, “2” and “3” denote the simulated case.

Typical profiles of density and temperature obtained from the simulation are plotted in Fig. 5. indicating low concentration of W particles (in different ionized state). To estimate the concentration of W ions we consider the “W-related” Z-effective:

(6) $${\text{Z}}_{\mathrm{eff}}^{\text{W}}=\frac{\sum_{\text{D,W}}z_{i}^{2}n_{i}}{\sum_{\text{D,W}}z_{i}n_{i}}\text{,}$$

which is plotted in Fig. 6. As we see, the concentration of W ions at the distances more than 1 cm from the divertor plates is negligibly small. Moreover, with the given resolution, $n_{\min}∼10^{15}\;{\text{m}}^{-3}$, we do not observe W ions with ionization state more than 4.

These results can be explained after analyzing of divertor plasma parameters from Table 1. As we can see, with increasing upstream temperature increases potential drop across the divertor plasma. As a result the electric field towards the divertor increases too and more W ions are attracted back to the plates (cf. the cases 1 and 2). Simulations indicate that the majority of the W atoms are ionized in the vicinity of the divertor plates and “promptly redeposited” there (see Fig. 6). Moreover, as we see from Fig. 6, the majority of W ions escaping prompt redeposition do not propagate far away from the divertor plates: they are kept within ∼1 m from the divertors and return back due to friction with main ions. As a result, only a very tiny fraction of sputtered W can escape reabsorbtion and propagate to the upstream SOL. The corresponding fluxes are given in Table 1.

**Table 1:** Plasma parameters at the divertors. First and second values correspond to the inner and outer divertors, respectively. $F_{\text{W}}^{\text{div}}$ and Δ*φ* denote the W ion flux density to the divertor plates and the potential drop across the divertor plasma. $F_{\text{W}}^{\text{upstrem}}$ denotes the sum of following W fluxes: the flux towards the upstream SOL and outflow at the radial boundaries due to W-atom radial transport and anomalous W-ion diffusion.

Case/value	1	2	3
$n_{e}\times 10^{19}\;{\text{m}}^{-3}$	4.5, 3.3	3.4, 3.5	3.2, 2.9
*T*_e_ eV	37, 53	20, 38	47, 58,
ΔφV	145, 176	88, 132	175, 188
$F_{\text{W}}^{\text{div}}\times 10^{20}\;{\text{m}}^{2}/\text{s}$	5.9, 3.5	5.1, 3.1	3.3, 3.8
$F_{\text{W}}^{\text{upstrem}}\times 10^{18}\;{\text{m}}^{2}/\text{s}$	6.6, 5.3	7.5, 3.6	4.6, 5.9

There is no significant contribution of hot C^++^ ions originating from the upstream SOL, because they are cooled down before reaching the divertor plasma.

## 4 Conclusions

Our simulations confirm experimental observations that W net erosion represents only tiny fraction (in our simulation ∼1%) of the W gross erosion. The estimated upstream W fluxes, $F_{\text{W}}^{\text{upstrem}}$, are in good agreement with the experimentally observed values $⩽10^{19}\;{\text{m}}^{-2}\;{\text{s}}^{-1}$ [16]. Moreover, this value is not very sensitive to the divertor plasma temperature. For low temperatures the energy of D and C ions hitting to the divertor plates is too low to sputter sufficient amount of W. With increasing energy the W sputtering increases, but the potential drop in the divertor plasma increases too. As a result, most of the W atoms are ionized in the vicinity of the divertor and return back to the plates. There are two effects leading to the observed prompt redeposition of W ions: first is the “near-divertor” ionization of W due to low ionization potential −7.86 eV (for comparison the ionization potentials for D and C are 13,6 and 10.6 eV), second, W^+n^ ions have large Larmor radius ∼2/nmm, so that they are redeposited within the distance of a Larmor radius. Important to note that a significant fraction of W ions escaping this prompt redeposition are returned back due to the friction with the main ions.

Our simulations indicated that the accuracy of $n_{\min}∼10^{15}\;{\text{m}}^{-3}$ is not sufficient for studying of the distribution of W charge states in the upstream SOL. These will be addressed in our future work.

## References

[1] Tskhakaya D., Jachmich S. (2011). J. Nucl. Mater..

[2] Coster D.P. (2011). J. Nucl. Mater..

[3] Takizuka T., Shimizu K. (2009). Nucl. Fusion.

[4] Havlickova E., Fundamenski W. (2012). Plasma Phys. Contr. Fusion.

[5] Tskhakaya D. (2012). Cont. Plasma Phys..

[6] Braginskii S.I., Leontovich M.A. (1965). Transport processes in a plasma.

[7] Alexandrov A.F., Bogdankevich L.S., Rukhadze A.A. (1984). Principles of Plasma Electrodynamics.

[8] D. Tskhakaya, A. Sobba, et. al., in: Proceedings of 18th Euromicro Conference on Parallel, Distributed and Network-based Processing, Pisa Italy, IEEE, 2010, 476.

[9] J. Bohdansky, Nucl. Fusion, 1984, p. 61 (special issue).

[10] Eckstein W. (2007). Top. Appl. Phys..

[11] Stenke M., Aichele K. (1995). J. Phys. B: At. Mol. Opt. Phys..

[12] Vainshtein L., Beigman I. (2011). J. Phys. B: At. Mol. Opt. Phys..

[13] C.D. Boley, J.N. Brooks, Y.-K. Kim, Rep. Argonne Natl. Lab., ANL/FPP/TM-171, 1983.

[14] Vörtler K., Björkas C., Nordlund K. (2011). J. Phys.: Condens. Matter.

[15] W. Eckstein, IPP Report, IPP 9/132, 2002.

[16] G.J. van Rooij, This Conference.

